# Cerebellar preference of luminal A and B type and basal ganglial preference of HER2-positive type breast cancer-derived brain metastases

**DOI:** 10.3892/mco.2021.2337

**Published:** 2021-06-30

**Authors:** Nobuyuki Izutsu, Manabu Kinoshita, Tomohiko Ozaki, Mio Sakai, Katsuyuki Nakanishi, Takahiro Nakayama, Yasuhiro Tamaki, Haruhiko Kishima

**Affiliations:** 1 Department of Neurosurgery, Osaka International Cancer Institute, Osaka 541-8567, Japan; 2 Department of Neurosurgery, Osaka University Graduate School of Medicine, Suita, Osaka 565-0871, Japan; 3 Department of Neurosurgery, Kawachi General Hospital, Higashiosaka, Osaka 578-0954, Japan; 4 Department of Diagnostic and Interventional Radiology, Osaka International Cancer Institute, Osaka 541-8567, Japan; 5 Department of Breast and Endocrine Surgery, Osaka International Cancer Institute, Osaka 541-8567, Japan

**Keywords:** breast cancer, brain metastasis, biological subtype, spatial distribution

## Abstract

The purpose of the current study was to investigate the hypothesis that the spatial distribution of brain metastases could be affected by the biological subtypes of breast cancer. CT (n=1) or MRI (n=66) images of 67 patients with a total of 437 treatment-naive brain metastases from breast cancer were retrospectively reviewed. Patients were grouped according to the biological subtype of the tumor [luminal A, 28; luminal B, 9; human epidermal growth factor receptor 2 (HER2) positive, 14; triple-negative breast cancer (TNBC), 16]. All images were standardized to the human brain MRI atlas provided by the Montreal Neurological Institute 152 database. The distribution pattern of brain metastases after image standardization was analyzed. The cerebellum and the frontal lobe were more commonly affected by breast cancer brain metastases. Brain metastases from luminal A and B types of breast cancer arose more often in the cerebellum. Brain metastases from HER2-positive type breast cancer occurred more often in the putamen and the thalamus and less frequently in the cerebellum than other types (P=0.0057). The subtypes of breast cancer are related to differences in the spatial distributions of their brain metastases. These differences may be utilized to plan different cranial irradiation strategies according to the breast cancer subtypes.

## Introduction

Brain metastases (BMs) are the most prevalent malignant tumors of the CNS. More than 200,000 patients are diagnosed with harboring BMs in the United States each year, and the number is more than 10-fold of the primary CNS tumors (1). Breast cancer is the second most frequent cause of BMs after lung cancer, and BMs occur in 24.6% of patients with metastatic lesions (2). The reported median survival time from diagnosis of BM ranges from six to 23 months (3). BMs are one of the major causes of systemic cancer-related mortality. Lately, the frequency of BMs is increasing due to advancements in treatment for primary cancers and in imaging techniques (4,5). On the other hand, the heterogeneity of BMs has been consistently reported since the 1950s. While such studies were performed postmortem in early years, image analysis with CT or MRI has recently become the primary research modality for this kind of study. Preferential involvement of the anatomic ‘watershed areas,’ the gray-white matter junction and the cerebellum, has been demonstrated in the development of BMs, and the relationship between the spatial distribution of BMs and primary cancer type has been discussed (6-9). Few reports, however, have discussed the relationship between the spatial distribution of BMs and biological subtypes of breast cancer.

In this report, the authors hypothesized that the spatial distribution of BMs could be affected by the biological subtype of tumors. To test this hypothesis, the authors compared the spatial distributions of breast cancer BMs and biological subtypes.

## Materials and methods

### 

#### Patients and data collection

This study was conducted in accordance with the Declaration of Helsinki, and the internal review board of the Osaka International Cancer Institute approved the clinical data used in this research. The authors retrospectively reviewed CT or MRI of radiation- and operation-naive breast cancer BMs treated at Osaka International Cancer Institute from 2008 to 2017, totaling 87 patients. Patients who underwent systemic chemotherapy, hormone therapy, molecular targeted therapy with agents such as trastuzumab, pertuzumab, or lapatinib, or radiation therapy were included. However, patients who underwent previous neurosurgical resection or brain radiation therapy of the lesion were excluded. Furthermore, the following patients were excluded from analysis: Five patients due to preexisting malignant diseases, three patients lacking a sufficient immunohistochemical profile of their breast cancer subtype, and 12 patients due to CNS lesions confined to the meninges, the dura, or the skull. As a result, BMs of 67 patients using contrast-enhanced CT, gadolinium-enhanced MRI T1-weighted images (1.5 or 3.0T), or plain T2-weighted images were included for analysis (1 by CT, 39 by 1.5T MRI, 27 by 3.0T MRI; 26 by thin slice with <1.5 mm thickness, 41 with 2-7 mm thickness).

#### Biological subtypes of breast cancer

Patients were divided into four groups according to the biological subtype of their breast cancer. Selection criteria were based on the expression of hormone receptors (HR) such as estrogen receptor (ER) and progesterone receptor (PgR), as well as human epidermal growth factor receptor 2 (HER2). Classified subtypes were as follows: HR-positive and HER2-negative (i.e., ER^+^ and/or PgR^+^, HER2^-^; luminal A), HR-positive and HER2-positive (ER^+^ and/or PgR^+^, HER2^+^; luminal B), HR-negative and HER2-positive (ER- and PgR^-^, and HER2^+^; HER2), and HR-negative and HER2-negative (ER^-^ and PgR^-^, and HER2^-^; triple-negative breast cancer; TNBC). Positivity of ER, PgR, and HER2 was evaluated via immunohistochemistry of the primary breast cancer or the BM (66 by primary cancer and one by BM). The threshold for a positive test was set at 1% or greater of cells being positive for ER or PgR. Fluorescence *in situ* hybridization analysis of HER2 amplification was carried out for cases where immunohistochemistry showed higher than 2+ for HER2. The authors also reviewed medical records to identify the initial symptoms of breast cancer BMs that led to performing brain imaging and compared these symptoms and breast cancer subtypes.

#### Image registration and frequency map reconstruction

**Figure 1:**
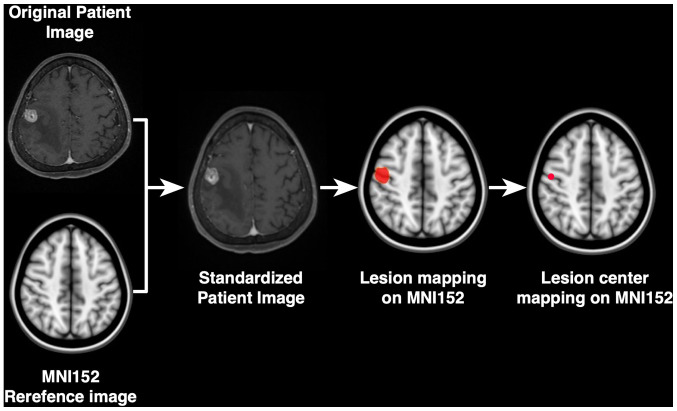
Schematic illustration of flow of image analysis. Each patient's brain MRI or CT was deformed and registered to standard brain MRI (MNI152) using FMRIB software library/FMRIB linear image registration tool. The lesions were manually identified. Thereafter, the center of gravity of the lesion was calculated automatically for each lesion, and voxel was defined as the primary site of occurrence.

All Digital Imaging and Communications in Medicine (DICOM) images were converted to Neuroimaging Informatics Technology Initiative (NIfTI) format using dcm2nii software (http://www.cabiatl.com/mricro/mricron/dcm2nii.html). These NIfTI data were registered to a 1.0-mm isotropic, high-resolution, T1-weighted brain atlas provided by the Montreal Neurological Institute (MNI152 database) using a mutual information algorithm with a 12-degree of freedom transformation using the Functional Magnetic Resonance Imaging of the Brain (FMRIB) Software Library/FMRIB Linear Image Registration Tool (FSL-FLIRT; http://fsl.fmrib.ox.ac.uk/fsl/fslwiki/FSL) (Fig. 1). Image registrations were visually confirmed in all cases before using FSL-FLIRT (10,11).

**Figure 2:**
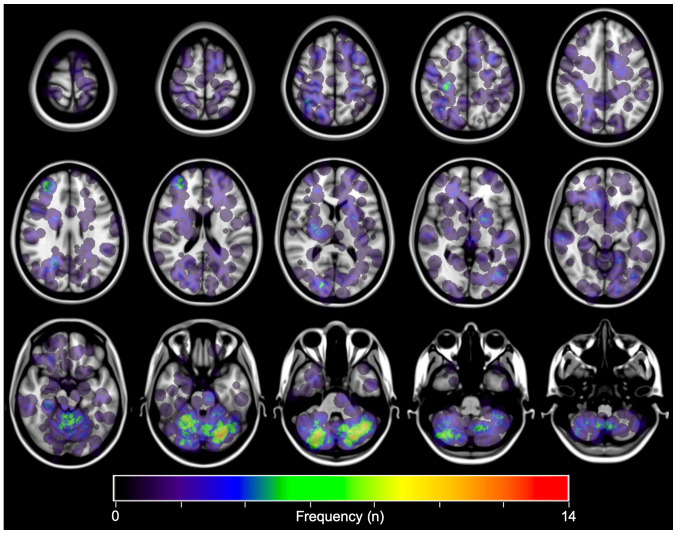
Axial image of the frequency map of all brain metastases from breast cancer. Note that the lesions are mostly concentrated in the cerebellum.

All datasets were exported to in-house software written in MatLab 7.14 (MathWorks) for further analysis. The lesions were manually identified. Thereafter, the center of gravity of each lesion was calculated automatically, and that voxel was defined as the primary site of occurrence. For frequency map reconstruction, all lesions were reconstructed to a site-centered spherical shape with a diameter of 20 mm. A heat map for the lesion occurrence frequency was reconstructed and superimposed on the reference MNI152 (Fig. 2). The voxel corresponding to the center of gravity of the lesion registered to the MNI structural atlas (12) was assigned to 11 segments as follows: Brainstem, caudate, cerebellum, frontal lobe, occipital lobe, parietal lobe, temporal lobe, pineal body, pituitary gland, putamen, and thalamus. As a result, each lesion was assigned to a specific anatomical region on the MNI152 structural atlas.

#### Statistical analysis

Statistical analysis was performed by JMP 14 (SAS Institute Inc.) and R version 3.5.2 (The R Foundation, Vienna, Austria). All P-values were two-sided, and P-values <0.05 were considered statistically significant. Data were compared between groups using the Chi-square test with residual analysis and the Kruskal-Wallis test for categorical and continuous variables. If not otherwise indicated, data are shown as medians with an interquartile range.

## Results

### 

#### Frequency map of metastatic brain lesions from breast cancer

In total, the authors analyzed 437 lesions from 67 patients as follows: 162 lesions from 28 patients with luminal A subtype, 30 lesions from 9 patients with luminal B subtype, 196 lesions from 14 patients with HER2 subtype, and 49 lesions from 16 patients with TNBC. Detailed characteristics of the patients are shown in Table I. There were no significant differences in the median numbers of BMs, the interval time from initial diagnosis of the primary lesion to development of BMs, and the ratio of patients with multiple BMs among biological subtypes.

MRI from 66 patients and CT from one patient were successfully transformed and registered on MNI152. The coordinates corresponding to each lesion's cancer were identified, with all lesions converted to spheres with a 20 mm diameter on MNI152. As can be appreciated in Fig. 2, breast cancer BMs most commonly involved the posterior fossa. This observation was consistent with past studies (6,9,13,14).

#### Different intracerebral distribution of breast cancer BMs by biological subtype

**Figure 3:**
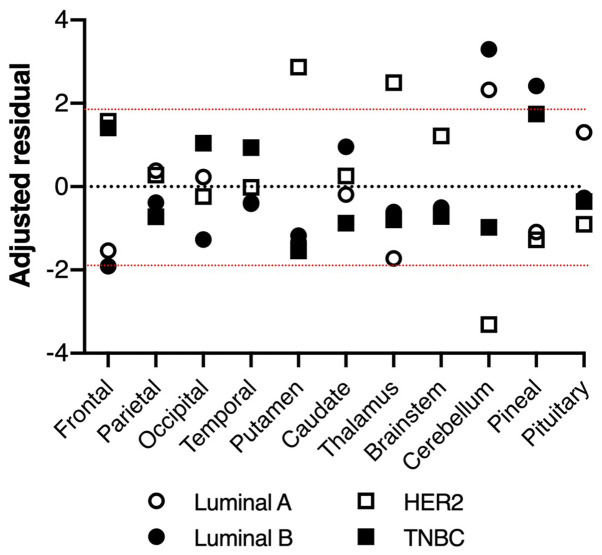
Upper dotted line indicates 1.96 of adjusted residual and lower dotted line -1.96. The plotted points beyond the upper line or below the lower line indicate statistical significance (P<0.05).

There was a significant difference in BMs' intracerebral distribution among subtypes using the Chi-square test (P=0.0057, Table II). Furthermore, the residual analysis revealed that, while BMs from luminal A and B type breast cancer predominantly occurred in the cerebellum with higher proportions than those from the other subtypes, BMs from HER2 breast cancer occurred in the cerebellum with a lower proportion and in the putamen and thalamus with higher proportions than those from the others (Fig. 3). Regarding the TNBC subtype, there were no significant characteristics in common with the other subtypes.

#### Anticancer drug therapies before BM occurrence

Most of the patients underwent multiple treatment regimens and various anticancer drugs were administered to each patient before their BMs were discovered. Three patients did not undergo any anticancer drugs because BMs had already occurred at the initial diagnosis of primary breast cancer. Detailed past treatment histories were not available for another three patients from outside institutions. We compared the distributions of BMs between two groups with and without chemotherapies, hormone therapies, and molecular targeted therapies for 431 BMs from 64 patients. There was no statistical significance in these comparisons with P-values being 0.43, 0.13, and 0.09, respectively (Chi-square test).

#### Symptoms at brain metastasis identification

**Figure 4:**
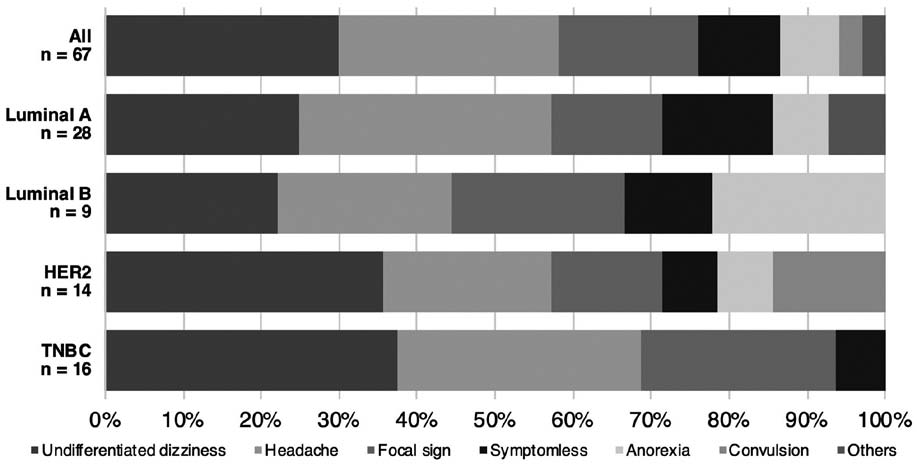
The proportion of symptoms at the time of brain imaging. There was no statistical significance.

The most frequently encountered clinical symptom was undifferentiated dizziness (29.9%), followed by headache (28.4%), and focal signs (17.9%) such as motor aphasia, hemiparesis, and visual disturbances. Seven patients did not present any neurological symptoms (10.4%). There was no significant difference in symptomatology among different breast cancer subtypes (P=0.51, Fig. 4).

## Discussion

Several classifications of biological subtypes based on their clinical, histopathological, and molecular characteristics are used for breast cancers. Furthermore, breast cancers can be grouped by their molecular expression states into luminal A or B, HER2, and triple-negative, and each subtype has different prognoses (15,16). TNBC and HER2 breast cancers are liable to develop BMs (2,3,17-19). Of note, the onset of BMs in TNBC is earlier, and overall survival is notably shorter than other subtypes (2,3). Anatomical watershed areas such as the gray-white matter junction are known as common sites for BMs (7). It is known that the spatial distribution of BMs are affected by the biological features of tumors. For example, the authors have recently reported that epidermal growth factor receptor (EGFR) mutated lung cancer BMs occurred more frequently in the caudate nucleus, cerebellum, and temporal lobe than those with an EGFR exon 19 deletion (8).

The primary purpose of the current study was to evaluate the spatial distribution of BMs from breast cancer in a more objective manner than past reports by using a voxel-based lesion mapping technique. In line with previous reports, the predominance of breast cancer BMs in the posterior fossa was visually confirmed (6,9,13,14). It is speculated that this tendency is due to the difference in blood flow between the cerebrum and the cerebellum (20). This study investigated the correlation between the spatial distributions of breast cancer BMs and their subtypes. The authors ascertained that BMs from luminal A and B type breast cancers concentrated in the cerebellum and these proportions were higher than those of the other subtypes, while HER2 breast cancer BMs occurred more frequently around the basal ganglia and less frequently in the cerebellum than the others. This tendency was inconsistent with previous reports as it was reported that BMs from HER2 positive and luminal type occurred dominantly in occipital lobe and cerebellum (21).

Kyeong *et al* reported that BMs from luminal type breast cancers were less frequently encountered in the cerebellum and that BMs from the TNBC subtype were more frequently encountered in the frontal lobe. This inconsistency with our findings may have been caused by differences in the segmentation of brain regions and the classification of biological subtypes. In the report by Kyeong *et al*, the whole brain was divided into 116 segments using the standardized automated anatomical labeling (AAL) template (21,22). Thereafter, those segments were rearranged into seven regions before analysis. This is a different segmentation scheme than employed in this study. Furthermore, Kyeong *et al* assigned patients with BMs from breast cancer to three groups: Luminal (HR-positive and HER2-negative), HER2 (HER2-positive and HR-positive or HR-negative), and TNBC (HR-negative and HER2-negative). This is different from the classification scheme of the current report. Further study is required to evaluate these differences.

Different metastatic patterns among breast cancer subtypes or among primary cancer entities were reported (23,24). Although the mechanisms causing differences of metastatic patters are not completely clarified, the ‘seed and soil hypothesis’ proposed by Paget is well accepted (25). The hypothesis assumes that compatibility between the biological characteristics of the cancer cells (‘seeds’) and the microenvironment of the metastatic site (‘soil’) plays an important role in cancer metastasis. Some key molecules for this phenomenon in breast cancer metastasis have been reported (26,27).

Regarding symptoms at the time of BM diagnoses, dizziness was the most frequently encountered neurological deficit. This finding could be related to the cerebellar distribution of BMs. On the other hand, screening for BMs was conducted only in 10% of patients as screening for BMs from asymptomatic breast cancer is not yet recommended. As the occurrence of BMs continues to increase in breast cancer patients (4,5), some reports propose clinical trials focusing on the value of BM screening in patients with breast cancer, particularly for subgroups at high risk of BMs (i.e., HER2 or TNBC with extracranial metastatic lesions) (28).

Regarding the treatment of breast cancer BMs, following treatment options are offered to the patients, which are surgical resection, whole-brain radiotherapy, stereotactic radiosurgery, chemotherapy, and targeted therapy (29,30). In addition, a treatment concept of prophylactic cranial irradiation (PCI) targeting breast cancer patients with high BMs risk has been proposed (31) similar to lung cancer. A randomized controlled trial evaluating the efficacy of PCI for high-risk breast cancer patients was performed, which revealed that patients receiving PCI did not develop BMs, while 6.4% of patients not receiving PCI developed BMs (32). In this context, understanding the characteristics of BMs due to breast cancer subtype may add insight into improving treatment strategies against this diseased condition. This includes the possibility of prophylactic irradiation with dose modulation to sites where BMs preferentially occur. For example, our research suggested that HER2 breast cancer occurred less often in the cerebellum but slightly more often in the putamen and thalamus. This localized treatment would aim for a preventive effect while reducing side effects. Further research of this kind could suggest different radiation dose modulation strategies according to the different subtypes of breast cancer.

This study has some limitations. Firstly, most of the brain lesions were not resected. Therefore, pathohistological diagnoses of brain lesions and concordances of biological subtypes between brain lesions and primary lesions were not confirmed. Secondarily, because of the retrospective analysis and small sample number, we could not analyze the genetic mutations which occurring frequently in breast cancers or associated with cancer metastasis. Likewise, some patients were referred to our department form outside institutions requesting treatment of brain lesions. Therefore, we could not calculate the true incidence of metastasis analyze the correlation between the frequency of metastasis by subtype and the brain region.

As a conclusion, BMs from luminal A and B types occurred more often in the cerebellum, while brain metastases from HER2-positive type breast cancer occurred more often in the putamen and the thalamus, and less frequently in the cerebellum than other types. These findings suggest that spatial distributions of BMs from breast cancers are affected by their biological subtypes.

## Figures and Tables

**Table I:** Patient characteristics.

Characteristic	All	Luminal A	Luminal B	HER2	TNBC	P-value
Patients (n)	67	28	9	14	16	-
Female (n)	67	28	9	14	16	-
Age at diagnosis						0.59
Median (years)	56	57.5	59	52.5	56.5	
IQR	(44.5-65.5)	(43.3-67.3)	(51.0-64.0)	(38.3-61.0)	(48.0-70.3)	
Interval time from initial diagnosisto BMs						0.17
Median (months)	46	70	24	33	47	
IQR	(23.0-93.0)	(28.8-112.3)	(23.0-57.0)	(18.5-59.8)	(29.5-68.8)	
Total number of BMs (n)	437	162	30	196	49	-
Number of BMs, n, median (IQR)						0.26
Median (n)	2	3.5	1	2	2	
IQR	(1-5.3)	(1.8-6.0)	(1.0-4.0)	(1.0-11.0)	(1.0-3.3)	
Patients with multiple BMs, n (%)	43(64)	21(75)	6(33)	9(64)	10(63)	0.81

HER2, human epidermal receptor 2; TNBC, triple-negative breast cancer; BMs, brain metastases; IQR, interquartile range.

**Table II:** Distribution of brain metastases.

Location	All n=437 (%)		LuminalA n=162 (%)		LuminalB n=30 (%)		HER2 n=196 (%)		TNBC n=49 (%)	
Frontal lobe	107	(24.5)	33	(20.4)	3	(10.0)	55	(28.1)	16	(32.7)
Parietal lobe	69	(15.8)	27	(16.7)	4	(13.3)	32	(16.3)	6	(12.2)
Occipital lobe	44	(10.1)	17	(10.5)	1	(3.3)	19	(9.7)	7	(14.3)
Temporal lobe	38	(8.7)	13	(8.0)	2	(6.7)	17	(8.7)	6	(12.2)
Putamen	18	(4.1)	4	(2.5)	0	(0.0)	14	(7.1)	0	(0.0)
Caudate	6	(1.4)	2	(1.2)	1	(3.3)	3	(1.5)	0	(0.0)
Thalamus	5	(1.1)	0	(0.0)	0	(0.0)	5	(2.6)	0	(0.0)
Brain stem	4	(0.9)	1	(0.6)	0	(0.0)	3	(1.5)	0	(0.0)
Cerebellum	143	(32.7)	64	(39.5)	18	(60.0)	48	(24.5)	13	(26.5)
Pineal body	2	(0.5)	0	(0.0)	1	(3.3)	0	(0.0)	1	(2.0)
Pituitary gland	1	(0.2)	1	(0.6)	0	(0.0)	0	(0.0)	0	(0.0)

HER2, human epidermal receptor 2; TNBC, triple-negative breast cancer.

## Data Availability

The datasets used and/or analyzed during the current study are available from the corresponding author on reasonable request.
